# Skin lesion monitoring at slaughter on heavy pigs (170 kg): Welfare indicators and ham defects

**DOI:** 10.1371/journal.pone.0207115

**Published:** 2018-11-12

**Authors:** Mattia Bottacini, Annalisa Scollo, Sandra A. Edwards, Barbara Contiero, Martina Veloci, Vincenzo Pace, Flaviana Gottardo

**Affiliations:** 1 Department of Animal Medicine, Production and Health, University of Padova, Agripolis, Legnaro, Padova, Italy; 2 Swivet Research snc, Reggio Emilia, Italy; 3 School of Agriculture, Food and Rural Development, Newcastle University, Newcastle upon Tyne, United Kingdom; 4 Suivet snc, Reggio Emilia, Italy; 5 OPAS, Pig Farmer Association, San Giorgio, Mantova, Italy; Tokat Gaziosmanpasa University, TURKEY

## Abstract

In order to evaluate at the slaughterhouse external carcass lesions on heavy pigs (170 kg) as potential welfare indicators, and the prevalence of ham defects determining ham exclusion from Protected Designation of Origin (PDO) markets, 732 pig batches from northern Italy were monitored during a 12-month period, and then processed analysing the effect of slaughter season, overnight lairage, and production type. On the slaughter line, skin scratches were separately scored in the posterior region (defined as the area including the hind legs and the tail) and the anterior one (as the remaining area), while the whole carcass was examined for external hematomas. Chronic ear and tail lesions referable to the rearing phase, and bursitis were recorded as retrospective welfare indicators. The annual median prevalence of carcasses per batch with severe anterior scratches was 64% while 46.4% had severe posterior scratches. The highest autumn score for both skin scratches (*P* < 0.001) and traumatic ham defects (*P* = 0.005) is reflected in the positive correlation between severe posterior scratches and ham hematomas (*r*^*2*^ = 0.27; *P* < 0.001). Overnight lairage batches resulted in higher prevalence for scratches, while among ham defects only veining increased. Among binary records, only ear lesions were frequently recorded (annual median = 10%). A comparison analysis between pigs in and out of PDO circuit was performed to evaluate the variation related to the different genetics, showing differences for ear and tail lesions and for almost all the considered ham defects. The present study confirms that skin lesions represent a problem also for heavy pigs and that overnight lairage and season can affect their prevalence, with the associated possibility to give ham defects. Ear lesions are suitable to be used as retrospective welfare indicator, while tail lesions usage is nowadays limited by the extensive use of tail docking.

## Introduction

Abattoir veterinary inspection has the main function of ensuring food safety, but scoring of visceral lesions (e.g. lungs, pleura, liver and stomach) can be also a useful tool for animal health monitoring and a source of data for epidemiological investigation [1–3]. Since animal welfare is of increasing interest for consumers and the wider society, measures providing retrospective information about the quality of the life of the animal during the rearing cycle are increasing in importance for the market. For this reason, additional abattoir measures are desirable to integrate with welfare assessments. In particular, monitoring of skin and tail lesions in pigs at the slaughterhouse has been proven to be a useful tool for the assessment of health and welfare on farm, highlighting their potential use as iceberg indicators [4], which had already been suggested for use as warning signals for welfare problems [5]. In this regard, abattoir inspections could contribute to enhance pig welfare standards by providing feedback to the farmer, who can then adopt appropriate interventions to improve on-farm prevention of these lesions, also reducing losses through lower rates of carcass condemnations, trimming and downgrading [6].

In Italy the requirements for the production of Protected Designation of Origin (PDO) hams result not only in an extended fattening period, with animals that are slaughtered at about 160–170 kg live body weight and at least 9 months of age, but also in well-defined carcass and ham trait requirements. The animals used must be purebred pigs of the basic traditional Large White and Landrace breeds or animals derived from those breeds, as well as improved types as listed in the Italian Herd Book. Animals derived from Italian Duroc are admitted too. These prescriptions in genetics, final body weight and cycle length should result in any case in a minimum requirement for thickness of visible cover fat on the thigh (20 mm for thighs up to 9 kg, and 30 mm for those over 9 kg), which is essential for PDO ham processing.

These PDO production schemes, which are designed to produce typical charcuterie products, have high quality standards and only hams without defects (as codified by Consortia of Parma and San Daniele ham) are admitted; as a result carcass lesions have a higher economic impact than in other production systems (PDO thighs have a higher value between 20% and 40% than non-PDO ones according to market variation). Defects of traumatic origin on the ham, resulting in both the exclusion from PDO market and welfare compromise, include hematomas, signs of bites and other lesions which make trimming necessary, whilst carcasses altered by stressful conditions and fighting before slaughter (e.g. pale, soft and exudative meat) can also undergo downgrading.

As many ham defects are caused by trauma, most of skin lesions detected at abattoir inspection are of traumatic origin. These traumatic skin lesions are commonly due to fightings, which occur when different pig groups are mixed together, as it happens before or after loading for transport to the slaughterhouse. Furthermore, stress caused by transport [7], fasting, prolonged lairage time [8] and environmental conditions [9] can negatively affect pigs’ behaviour, resulting in increased skin damage.

The heavier slaughter weight of Italian pigs destined to PDO productions may pose an additional risk, making skin lesions more prevalent due to the attainment of sexual maturity in females, as well as the progressive decrease in space allowance in a barren environment: EU Council Directive 2008/120/EC fix minimal space requirements generally for pigs over 110kg, leading to a legal vacuum for much heavier weights.

Over recent years, an increasing demand for higher slaughter weight pigs out of PDO schemes (for the purpose of fresh meat production) is emerging in Italy. In this production type, no breed restrictions are present (which are present for pigs destined to PDO production), so farmers can use the genetics with the best growth performance. Despite a shorter rearing cycle due to the faster daily growth and different carcass traits (more lean carcass), this productive type is also slaughtered at the final weight of 170 kg. The different genetics used for heavy pigs in or out of PDO circuit could therefore represent another variable affecting the prevalence of external carcass lesions and ham defects.

The aim of this work was to describe the frequency of different external lesions, which are suitable to be used as welfare indicators, during slaughtering procedures of 170 kg live weight pigs, and the prevalence of ham defects detected on the day after slaughter, considering the effects of season, overnight lairage and animal type (effect of genetic). The collected data were then used to evaluate possible relationships between fighting lesions on the carcass and ham defects.

## Materials and methods

### Collection of data

Data collection was carried out for 12 months from January to December 2016, through a weekly monitoring on all the batches (one batch per farm per day) slaughtered on Mondays, for a total of 49 sampling days, in an abattoir located in Emilia Romagna (Società Cooperativa Agricola OPAS–Organizzazione Prodotto Allevatori Suini) with a slaughtering capacity of 5,000 fatteners per day.

All pigs were transported from farm to the abattoir by trucks in batches of about 135 (minimum 130; maximum 140) animals. All pigs belonging to the same batch were derived from the same farm and were consecutively slaughtered on the same day. Batches unloaded in the morning were slaughtered on the day of arrival, while batches unloaded in the late afternoon had a longer lairage time since slaughter was performed on the following morning starting at 5 a.m. In each batch, composed of both females and barrows, about 100 animals (minimum 95; maximum 105) were randomly selected in the middle of the batch for the external carcass evaluation, omitting those at the beginning and end of each batch in order to avoid any risk of accidental inclusion of pigs belonging to the previous or the following batch. The identification of each batch was provided by reading codes tattooed on the skin of hams for Italian heavy pigs and by ear marks or tattoos for pigs not labelled on the ham. The study involved 648 batches of Italian heavy pigs destined to PDO production and 84 batches of non-PDO (out of the PDO scheme heavy pigs), resulting in about 64,800 Italian heavy pigs destined to PDO and 8,400 heavy pigs out of the circuit, from 267 intensive fattening farms located in the North of Italy, with no different prescriptions for facilities between the two different production types.

### Carcass inspection

Slaughter line speed was 480 animals per hour and inspection of the carcass was performed directly during the slaughtering process from a designated position on the line after scalding and before de-hairing of the carcass, which was still entire at the time of evaluation. The same veterinarian, previously trained for 5 weekly whole-day scoring sessions to develop good intra-observer reliability, always conducted this inspection. Examination of external carcass lesions was conducted by a visual inspection, recording the scores directly into an Excel file using a tablet (http://dx.doi.org/10.17504/protocols.io.ugketuw [PROTOCOL DOI]).

### Fighting and transport lesions

**Fig 1:**
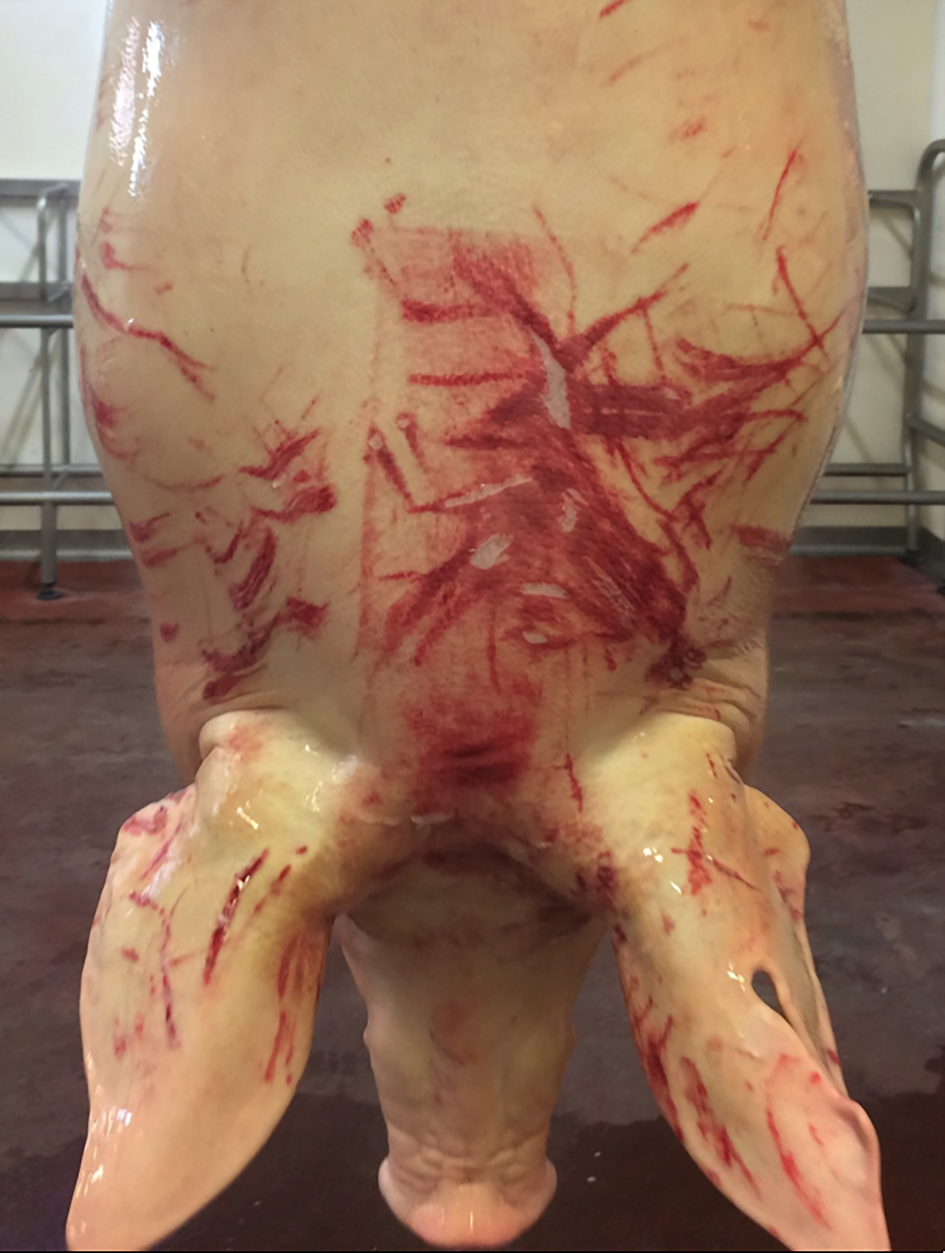
Anterior part of the carcass. An example of score 2.

To score acute traumatic lesions (scratches), the carcass was divided into two parts: the “posterior” region, which included the hind legs and the tail, and the “anterior” region defined as the remaining area (starting from the loin up to the front limbs, the head and the ears). The decision to consider only the thigh as the “posterior” region was driven by its economic relevance in the Italian PDO production. In order to easily scan the carcasses during their rapid passage on the dressing line (480 pigs/h), a 3 point scoring system for each of the two carcass regions was used: score 0, up to one scratch or bite; score 1, from two to five scratches or bites; score 2, more than five scratches or bites or any wound which penetrates the muscle (Fig 1) (similarly to the Welfare Quality Protocol, which differs both for the perimeter of the regions and for the number of scratches per score [10]). In addition, external hematomas were recorded as presence or absence (binary score).

### Lesions providing information for retrospective welfare assessment

The presence of lesions with a chronic onset such as notches, necrosis, bites and scars were also evaluated on ears and tail as welfare indicators [4] referable to the rearing cycle, as well as the presence of bursitis. Bursitis was only evaluated on the forelimbs, because hind legs were not completely visible to the observer since the carcass was hung upside down above the inspection area. These lesions were recorded as present or absent (binary score) regardless of the size, in accordance with the possibility to be detected in the few seconds allocated to the observer. For ear and tail lesions, only the older ones were recorded as retrospective welfare indicators, since the recent ones were already recorded as pre-slaughter fighting or transport lesions, so that recent hyperaemic or fresh bleeding lesions were not recorded as retrospective indicators. Post-mortem artefacts due to the slaughtering process were excluded on the basis of the absence of pathological or physiological changes occurred in-vivo such as, for example, necrosis or scarring.

### Ham defects

On the day after slaughter, all the separated thighs, from here named hams, were evaluated according to PQI (Parma Quality Institute) standards [11] and defect detection was performed on each ham belonging to the batches previously scored for skin lesions. Only traumatic (hematomas, muscular lacerations, tendon-bone lacerations) and stress-related defects (pale soft exudative meat, petechial haemorrhaging, veining) were considered in this study. All the considered defects are causes for ham exclusion from the PDO market. In the case of more than one defect on the same ham, only the one causing the highest percentage of trimming or affecting the whole ham was considered, so that in the dataset one ham corresponded to one defect. Ham traceability was ensured by matching batch identification numbers and ham codes (tattooed on farm or labelled during the slaughtering process).

### Statistical methods

All the statistical analyses were performed in SAS (Inst. Inc., Cary, NC). For each batch, the prevalence for every score was calculated, as well as the frequency of binary variables. To analyse the sources of variation on outcome variables, lairage duration, season of slaughtering and farm of origin were considered as explanatory factors. Farm of origin was included as random effect into the model, which was corrected for it in order to remove this source of variation when analysing the other factors. A further analysis, including farm of origin as fixed effect, was performed. Based on the order of the slaughter, it was possible to identify the batches of pigs that had been subjected to overnight lairage (the first 10 of the daily list), so as to take account of the prolonged fasting (not more than 12 h) and lairage time (at least 8 h). The lairage time effect was dichotomized into two levels: delivery day slaughter *vs* overnight lairage. Season of slaughtering was categorised according to the equinoxes and solstices as occurred during 2016: autumn (22 September–20 December), winter (21 December–19 March) spring (20 March–19 June), summer (20 June–21 September). Data were analysed for their distributions (PROC UNIVARIATE). For normally distributed data an ANOVA was carried out using season, lairage and their interaction as the fixed effects and farm as random effect (PROC MIXED). For non-normally distributed data a log (x+1) transformation was applied and, if the normality was still not achieved, non-parametric tests were used: Kruskall-Wallis and Mann-Whitney respectively for season and lairage effects (PROC NPAR1WAY). The relationships between the prevalence and scores of the different lesions were assessed at the batch level using Spearman’s rank correlation (PROC CORR). Both the ANOVA that considered seasonality, lairage duration and farm of origin and the Spearman’s rank correlation were performed on 648 batches of Italian heavy pigs destined to PDO.

A further analysis was performed to compare Italian heavy pigs destined to PDO and heavy pigs out of the circuit. Since only 2 non-PDO pig batches were sampled during winter all the batches slaughtered in that season (PDO and non-PDO) were excluded from this further analysis. Non-PDO pigs were always slaughtered on the day of arrival, so all the overnight lairage batches were also excluded from the model. A total of 256 batches of Italian heavy pigs destined to PDO and 82 of heavy pigs out of the circuit were consequently considered. The analysis was conducted using a parametric or non-parametric approach for normally or not-normally distributed data respectively.

## Results

Data process did not show significative interactions between the season and lairage effects, therefore in the tables are shown only the main effects.

### Prevalence of lesions on the carcass

The prevalence for each external lesion observed on the carcass of 648 PDO batches is reported in Tables 1 and 2, in which variables affected by slaughter season and overnight lairage (respectively) are shown. The farm of origin effect was calculated when data distribution was normal and it explained 20% of variation in the ANOVA analysis. When farm of origin was included as a fixed effect in the model, the results of the statistical analysis were not different from those obtained using farm as a random effect (the reported one). Among binary lesions, only ear lesions occurred frequently, whereas others (tail biting, bursitis and hematomas) were more sporadic (< 2% prevalence).

**Table 1:** Effect of season on the prevalence of different skin lesions detected in 648 batches of PDO Italian heavy pigs.

	Annual (n = 648)	Spring (n = 163)	Summer (n = 161)	Autumn (n = 156)	Winter (n = 168)	SEM	*P*-value season	*F* or *K*	Farm effect (% variation)
**Fighting lesions:**									
Anterior region, score 2^1^ (%)	64.0 (0–100.0)	49.0^c^ (2.2–94.7)	74.7^b^ (0–100.0)	100.0^a^ (0–100.0)	23.0^d^ (0–100.0)		< 0.001	306.6	
Posterior region, score 2^1^ (%)	46.4±24.7	43.4^b^	49.0^b^	64.7^a^	28.5^c^	1.76	< 0.001	89.2	20.0
Hematomas (%)	0 (0–2.0)	0 (0–1.3)	0 (0–1.2)	0 (0–1.1)	0 (0–2.0)		0.50	2.4	
**Retrospective indicators:**									
Ear lesions^2^ (%)	10.0 (0–99.0)	14.0^a^ (0–97.0)	9.1^b^^c^ (0–80.0)	8.0^c^ (0–99.0)	10.8^a^^b^ (0–98.6)		< 0.001	26.7	
Tail lesions^2^ (%)	0 (0–41.0)	0^c^ (0–5.3)	0^a^ (0–10.0)	0^a^ (0–19.7)	0^b^ (0–41.0)		0.003	14.3	
Bursitis (%)	1.0 (0–10.0)	1.1^a^ (0–7.0)	1.0^a^^b^ (0–9.2)	1.0^b^^c^ (0–6.2)	1.0^c^ (0–10.0)		< 0.001	35.9	

The different lesion prevalences are expressed as the percentage of carcasses with that score or lesion within the batch. Annual and seasonal values are shown as median and range in brackets (non-parametric data, K: Kruskall-Wallis test) or LS-mean ± standard error (normally distributed data, F statistic reported) corrected for the effect of farm.

SEM: standard error of the mean.

Farm effect (% variation): the percentage of variation in the prevalence of a specific score or lesion referable to the farm of origin (reported only when it reached the statistical significance).

^1^: more than five scratches or any wound which penetrates the muscle

^2^: recent lesions were not considered.

^a,b,c,d^: values within the same row with different superscripts differ significantly (*P* < 0.05).

**Table 2:** Effect of lairage on the prevalence of different skin lesions detected in 648 batches of PDO Italian heavy pigs.

n = 648	Same day slaughter (n = 358)	Overnight lairage (n = 290)	SEM	*P*-value lairage	F or U	Farm effect (% variation)
**Fighting lesions:**						
Anterior region, score 2^1^ (%)	58.0^b^ (0–100.0)	70.2^a^ (0–100.0)		0.02	51703	
Posterior region, score 2^1^ (%)	44.8	48.0	1.59	0.09	2.9	20.0
Hematomas (%)	0 (0–2.0)	0 (0–1.1)		0.33	46211	
**Retrospective indicators:**						
Ear lesions^2^ (%)	9.0^b^ (0–99.0)	11.3^a^ (0–98.5)		0.01	52314	
Tail lesions^2^ (%)	0 (0–41.0)	0 (0–8.0)		0.32	48236	
Bursitis (%)	1.0^b^ (0–10.0)	1.0^a^ (0–9.2)		< 0.001	55852	

The different lesion prevalences are expressed as the percentage of carcasses with that score or lesion within the batch. The values for same day slaughter or overnight lairage batches are shown as median and range in brackets (non-parametric data, U: Mann-Whitney non parametric test) or LS-mean (normally distributed data, F statistic reported) corrected for the effect of farm.

SEM: standard error of the mean.

Farm effect (% variation): the percentage of variation in the prevalence of a specific score or lesion referable to the farm of origin (reported only when it reached the statistical significance).

^1^: more than five scratches or any wound which penetrates the muscle

^2^: recent lesions were not considered.

^a,b,^: values within the same row with different superscripts differ significantly (*P* < 0.05).

Statistical analysis showed that season had a strong effect on almost all the recorded lesions, except for external hematomas. Autumn season had the highest prevalence of scratches due to recent fighting, while spring was the season that overall had the highest values for lesions referable to the rearing cycle (*P* < 0.001), except for tail biting; this parameter had a flat seasonal trend (yearly average: 0.60%) as shown by the seasonal medians, apart from winter, when it reached its lowest values (*P* = 0.003). Winter was the season with the lowest prevalence of external carcass lesions.

Overnight lairage resulted in higher frequencies of scratches, expressed both as mean scratch score and prevalence of severe scratches (score 2) on the anterior part of the carcass (*P* < 0.05). Severe scratches in the posterior region showed the same trend but, together with hematomas, this did not reach statistical significance. Among retrospective indicators, ear lesions (*P* = 0.01) and bursitis (*P* < 0.001) had a higher prevalence in overnight lairage batches, while tail lesions were not significantly affected by lairage.

### Prevalence of ham defects

The seasonal variability of traumatic (ham hematomas, muscular lacerations, tendon-bone lacerations) and stress-related defects (PSE, petechial haemorrhaging, veining) is reported in Table 3. Autumn and spring resulted the seasons with the highest prevalence of traumatic ham defects (*P* = 0.005). Considering lairage (Table 4), only petechial haemorrhaging (reduced with overnight lairage; *P* < 0.001) and veining (higher prevalence with overnight lairage; *P* = 0.002) reached statistical significance. The farm effect explained 44% of the variation in the prevalence of traumatic-stress related defects (the superclass resulting from the sum of the two summary class: traumatic and stress related defects). There was no interaction between season and lairage effects.

**Table 3:** Effect of season on the prevalence of different ham defects detected in 648 batches of PDO Italian heavy pigs.

	Annually (n = 648)	Spring (n = 163)	Summer (n = 161)	Autumn (n = 156)	Winter (n = 168)	SEM	*P*-value season	*F* or *K*	Farm effect (% variation)
**Summary classes:**									
Traumatic defects (%)	4.7 (0–35.1)	4.8^a^^b^ (0.4–21.0)	4.7^b^ (0–21.8)	5.2^a^ (0.3–35.1)	4.3^b^ (0–18.0)		0.005	12.8	
Traumatic-Stress related defects (%)	8.7±4.7	9.8^a^	8.2^b^	9.9^a^	9.0^a^^b^	0.41	< 0.001	5.6	43.6
**Traumatic defects:**									
Hematomas (%)	3.3 (0–33.6)	3.6^a^^b^ (0.4–16.3)	3.2^b^^c^ (0–16.6)	3.8^a^ (0–33.6)	2.8^c^ (0–16.7)		< 0.001	17.8	
Muscular lacerations (%)	1.0 (0–9.4)	1.1 (0–9.2)	0.8 (0–7.6)	1.1 (0–9.4)	0.9 (0–9.0)		0.30	3.7	
Tendon-bone lacerations (%)	0 (0–2.1)	0^a^^b^ (0–1.1)	0^b^ (0–2.1)	0^a^^b^ (0–1.0)	0^a^ (0–2.1)		0.02	9.6	
**Stress related defects:**									
PSE (%)	0 (0–4.9)	0^a^ (0–3.6)	0^a^ (0–2.8)	0^a^ (0–4.9)	0^b^ (0–3.7)		< 0.001	22.0	
Petechial haemorrhaging (%)	0.7 (0–6.8)	0.7^a^^b^ (0–5.5)	0.8^a^ (0–6.8)	0.8^a^ (0–6.2)	0.4^b^ (0–3.6)		< 0.001	21.0	
Veining (%)	1.6 (0–20.7)	2.0^a^ (0–20.7)	1.0^b^ (0–12.5)	1.8^a^ (0–11.6)	2.1^a^ (0–19.1)		< 0.001	50.7	

Traumatic defects comprise hematomas, muscular lacerations and tendon-bone lacerations, while stress related defects comprise PSE, petechial haemorrhaging and veining.

Traumatic-Stress related defects result from the sum of these two classes.

The different defect prevalences are expressed as the percentage of hams with that defect within the batch (only the most extensive defect was considered in case of multiple defects on the same ham). Annual and seasonal values are shown as median and range in brackets (non-parametric data, K: Kruskall-Wallis test) or LS-mean ± standard error (normally distributed data, F statistic reported) corrected for the effect of farm.

SEM: standard error of the mean.

Farm effect (% variation): the percentage of variation in the prevalence of a specific defect referable to the farm of origin (reported only when it reached the statistical significance).

^a.b.c.d^: values within the same row with different superscripts differ significantly (*P* < 0.05).

**Table 4:** Effect of lairage on the prevalence of different ham defects detected in 648 batches of PDO Italian heavy pigs.

n = 648	Same day slaughter (n = 358)	Overnight lairage (n = 290)	SEM	*P*-value lairage	*F* o *U*	Farm effect (% variation)
**Summary classes:**						
Traumatic defects (%)	4.7 (0.4–15.4)	4.8 (0–35.1)		0.84	48922	
Traumatic-Stress related defects (%)	9.1	9.3	0.36	0.61	0.3	43.6
**Traumatic defects:**						
Hematomas (%)	3.3 (0–14.55)	3.3 (0–33.59)		0.34	50374	
Muscular lacerations (%)	1.0 (0–9.04)	1.0 (0–9.39)		0.63	49246	
Tendon-bone lacerations (%)	0^a^ (0–2,1)	0^b^ (0–1,5)		< 0.001	42620	
**Stress related defects:**						
PSE (%)	0 (0–4.9)	0 (0–3.7)		0.11	47136	
Petechial haemorrhaging (%)	0.8^a^ (0–6.8)	0.4^b^ (0–3.7)		< 0.001	33622	
Veining (%)	1.5^b^ (0–12.2)	2.0^a^ (0–20.7)		0.002	57448	

Traumatic defects comprise hematomas, muscular lacerations and tendon-bone lacerations, while stress related defects comprise PSE, petechial haemorrhaging and veining.

Traumatic-Stress related defects result from the sum of these two categories.

The different defect prevalences are expressed as the percentage of hams with that defect within the batch (only the most extensive defect was considered in case of multiple defects on the same ham). The values relating to same day slaughter or overnight lairage batches are shown as median and range in brackets (non-parametric data, U: Mann-Whitney non parametric test) or LS-mean (normally distributed data, F statistic reported) corrected for the effect of farm.

SEM: standard error of the mean.

Farm effect (% variation): the percentage of variation in the prevalence of a specific defect referable to the farm of origin (reported only when it reached the statistical significance).

^a,b,^: values within the same row with different superscripts differ significantly (*P* < 0.05).

### Correlation between carcass lesions and ham defects

No correlation over the 0.6 threshold was found between scratches on the carcass and ham defects. Only a low positive correlation between prevalence of severe scratches (score 2) on the thigh and hematomas detected by ham defect evaluation was found (*r*^*2*^ = 0.27; *P* < 0.001).

### Differences in the prevalence of skin lesions and ham defects between Italian heavy pigs destined to PDO and heavy pigs out of the circuit

The prevalence of nearly all ham defects (*P* < 0.001) and ear lesions (*P* < 0.001) were higher in PDO batches than non-PDO ones. Only tail lesions had a higher prevalence in non-PDO batches than in the PDO ones (*P* < 0.001). Results are shown in Table 5; the interaction between season of slaughtering and production type did not reach statistical significance.

**Table 5:** Differences in the annual prevalence of skin lesions and ham defects between 256 batches of heavy Italian pigs destined for PDO production and 82 batches of Non-PDO heavy pigs.

n = 338	non-PDO heavy pigs (n = 82)	PDO Heavy Pigs (n = 256)	SEM	*P*-value	*F* or *U*
**SKIN LESIONS:**					
**Fighting lesions:**					
Anterior region, score 2^1^ (%)	92.5 (19–100)	78.6 (1–100)		0.15	7772
Posterior region, score 2^1^ (%)	46.2	50.4	2.22	0.19	1.7
Hematomas (%)	0 (0–1)	0 (0–1.3)		0.89	8800
**Retrospective indicators:**					
Ear lesions^2^ (%)	4.7^b^ (0–33)	9.0^a^ (0–99)		< 0.001	10908
Tail lesions^2^ (%)	0.5^a^ (0–15)	0^b^ (0–19.7)		< 0.001	6529
Bursitis (%)	1.0 (0–6.7)	1.0 (0–7)		0.95	8683
**HAM DEFECTS:**					
**Summary classes:**					
Traumatic defects (%)	1.9^b^ (0–9.6)	4.8^a^ (0.4–15.4)		< 0.001	17251
Traumatic-Stress related defects (%)	3.8	9.1	0.41	< 0.001	79.9
**Traumatic defects:**					
Hematomas (%)	1.1^b^ (0–6.1)	3.5^a^ (0–14.5)		< 0.001	17899
Muscular lacerations (%)	0.7^b^ (0–6.1)	1.0^a^ (0–7.6)		< 0.001	13062
Tendon-bone lacerations (%)	0 (0–1.8)	0 (0–2.1)		0.43	9793
**Stress related defects:**					
PSE (%)	0^b^ (0–0.4)	0^a^ (0–4.9)		< 0.001	14773
Petechial Haemorrhaging (%)	0.2^b^ (0–2.6)	1.0^b^ (0–6.8)		< 0.001	16355
Veining (%)	0.4^b^ (0–3.5)	1.3^a^ (0–12.2)		< 0.001	14896

The different lesion (or defect) prevalences are expressed as the percentage of carcasses (or hams) with that score or lesion (or defect) within the batch. The values are shown as median and range in brackets (non-parametric data, U: Mann-Whitney non parametric test) or LS-mean (normally distributed data, F statistic reported).

non-PDO: non Protected Designation of Origin; PDO: Protected Designation of Origin.

SEM: standard error of the mean.

^1^: more than five scratches or any wound which penetrates the muscle

^2^: recent lesions were not considered.

^a,b^: values within the same row with different superscripts differ significantly (*P* < 0.05).

## Discussion

Fighting before slaughter, which takes place especially when unfamiliar pigs are mixed together during or after transport, is a very common negative behaviour for pig welfare resulting in carcass damage [12]. In this study a very high prevalence of scratches was found in every season, with a peak during autumn. When considering the anterior part of the carcass, in most cases nearly all the pigs recorded within a batch had severe scratches. This very high prevalence confirms how fighting is a very common behaviour in pigs under stressful conditions like mixing, transport, fasting and lairage [8]. Although we mainly refer to the phases immediately preceding the slaughter, it can’t be excluded that recent lesions could be occurred in the final rearing phase (especially for deep, bleeding lesions which could take more time to heal than surface scratches). The higher slaughter weight of pigs in Italy could be a risk for increasing fighting behaviour, since space allowance in both the farm and the lairage pen is regulated upon lighter weight pigs (1m^2^ for pigs over 110 kg as stated in EU Council Directive 2008/120/EC), but the use of different scoring methods for evaluation of scratches at the slaughterhouse makes it impossible to compare between different studies and pig production systems. Further studies on space allowance for these pigs, slaughtered at heavier weight compared to the European average, could highlight if this regulatory vacuum needs legislative specifications to ensure better minimum welfare standards for this category of animals.

A strong effect of season was found both on carcass lesions and ham defects, while the effect of the farm of origin seems to have had less influence on the processed variables (the normally distributed ones), probably because of the similar genetics and management conditions on intensive farms involved in Italian PDO production. Autumn was the season with the highest prevalence of recent fighting scratches, confirming the results of Gosàlvez et al. [13]. Barton Gade et al. [14] found that creatin kinase in serum is positively correlated to skin lesions, and the higher values of this enzyme detected during summer compared to winter samplings by Sommavilla et al. [15] (together with increased cortisol levels) is consistent with our results, since in the present study summer was the second highest season for anterior scratches. Correa et al. [16] found a higher prevalence of fighting and mounting type bruises in trials carried out during summer compared to the winter, although the overall skin damage score which resulted was higher during winter, which has been reported to be the season with the highest scores also in other studies [17]. This higher winter prevalence had been related to the more frequent standing behaviour during transport associated with cold temperatures, combined with the slipperiness of humid loading docks and trucks [18]. Although we found an increase of tendon-bone lacerations in hams also during winter, which could be related to more frequent slips, considering only fighting-related external lesions these studies seem to be in contrast with our results, since winter was the season with the lowest prevalence of scratches. However it should be noted that, in contrast to other studies, ours had a 12-month duration and several sampling days were performed in each season. Furthermore, mean seasonal temperatures between Italy and other countries where studies have been reported are very different, whilst the slaughtering weight of 170 kg in Italian heavy pig production might make animals less sensitive to cold temperatures, reducing the effect of temperature in cold seasons.

The attainment of puberty could also play a role in skin lesion frequency. The 9 months production cycle in Italian heavy pigs makes sex-related behaviours possible in the final finishing phase of female pigs (all males are surgically castrated in the first week of life) and different photoperiods could determine a delayed or an earlier attainment of puberty in non-stimulated female finishers. In a study considering entire male pigs, Prunier et al. [19] found more skin lesions on entire male pigs in autumn than in spring, attributing this effect to accelerated male pubertal development during autumn. The influence of photoperiod on puberty onset in gilts remains controversial; it seems to play a prominent role in infertility and delayed puberty [20], but some authors report an earlier age of first mating in gilts exposed to increasing photoperiod [21] while others support the role of a decreasing photoperiod in attaining earlier puberty [22].

Normally female pigs in Italian PDO production are slaughtered close to reaching full sexual maturity, which is normally achieved after the 7^th^ month of life in replacement gilts, and the behavioural effects of heat manifestation can often be observed. Therefore, the highest traumatic skin lesion prevalence on the carcass detected in pigs slaughtered during autumn might be explained by a greater heat manifestation in this season, with behaviours linked to sexual maturity being more evident in gilts which are at least 270 days old than in the younger and lighter weight animals in other production systems. Sexual hormones were, in fact, demonstrated to be related to more aggressive interactions and skin lesions at slaughter in intact females compared to immune-castrated heavy female pigs [23].

An overnight lairage, which is associated with a longer fasting time, resulted in an increased prevalence of scratches when looking at the mean carcass value, but in particular those on the anterior part, which indicate typical frontal and lateral aggressive fighting [16]. This confirms the results of Nanni Costa et al. [24] on Italian heavy pigs, who found that pigs held in overnight lairage showed a higher presence of severe skin damage in all carcass parts (values almost tripled), compared to shorter lairage. It is also well documented that longer periods of fasting result in increased pig aggressiveness [8]. In addition, due to the strict standardisation of management from loading at the farm (always performed by the same truck companies) to unloading at the abattoir (always performed by the same abattoir operators), it is unlikely that the higher presence of skin damage in overnight rested pigs occurred before lairage.

Among variables that more than the other ones provide information on animal welfare at farm level, only ear lesion prevalence was numerically relevant, reflecting a quite frequent on-farm problem of ear necrosis and/or ear biting. A strong seasonality was found, and winter and spring were the seasons with the highest ear lesion frequency. Since we are not able to precisely date the lesion onset, we can only suppose that this highest frequency could refer to lesions dated in these seasons or more probably to older ones dated in the cold periods (autumn and winter), which could present a higher risk for ear necrosis due to the higher air humidity [25]. The higher prevalence in overnight batches remains controversial, since only chronic lesions were recorded and overnight lairage is a variable occurring close to the observation time. Because ear lesions influence market value in certain regions and have the potential to be used as a retrospective welfare indicator, further analysis on the type and origin of these lesions is merited. The possibility of using ear lesions as iceberg indicator should be verified, correlating them with parameters recorded on-farm.

Tail biting lesions recorded during abattoir inspection have already been proposed, together with skin lesions, as potential iceberg indicators of pig health and welfare [4]. However their very low prevalence (median = 0) in each season, confirming the 0.18% of lesions found on-farm in weaning and fattening pigs by Scollo et al. [26], precludes their full potential for this purpose. Since the percentage of pigs with docked tails in Italian pig farms is close to 100% [27], tail lesions will not be very useful as an indicator in retrospective welfare assessment until this practice is changed, and used only as a last emergency solution after failure of any other preventive interventions. However, considering the growing percentage of undocked slaughtered pigs in the abattoir that hosted the observations (on a total of 750,000 Italian heavy pigs slaughtered per year: 0% in 2016; 6% in 2017; 10% expected in 2018), the potential of this indicator should be reconsidered in the future.

The prevalence of bursitis (at least one limb affected) has been reported in different studies to be very high: 41.2% on farm recorded on the four limbs [28]; 44% at abattoir recorded on the hind legs [29]. Older pigs have been reported to be at higher risk to develop this lesion because their greater body weight exerts additional pressure on the limbs and they spend a greater proportion of time lying [30], so we expected higher bursitis prevalence at slaughter in Italian heavy pigs. Nevertheless we detected a maximum batch value of only 10%, but it must be considered that we examined only the forelimbs, while bursitis is reported to occur more frequently on the hind legs [28]. In the present study, the median was around 1% in each season and, since our binary score (presence or absence) had to fit with other recordings in the few seconds allocated to the observer, a lower sensitivity might have resulted when compared to other targeted studies. With these results we can, however, hypothesize that heavier slaughtering weight could not determine higher bursitis prevalence than in pigs slaughtered around 110 kg.

As mentioned above, traumatic and stress-related ham defects had an overall high relative frequency, confirming that the number of rejected hams from cured ham production is still a relevant economic problem for the Italian pig industry. A seasonal effect on ham defects was evident, with autumn (as in the case for scratches) and spring resulting in the worst outcomes for traumatic defects. Considering this defect class, a seasonal overlap between carcass skin lesions and ham defects was clear in autumn, although there was only a low positive correlation within batches. There could have been an underestimation of the carcass damage due to the position of the observer on the slaughter line, which did not allow good visual detection of external hematomas on the thighs, which were often localized on the ventral part, as well as above the line of sight. The difference between hematomas found on the carcass during the slaughtering process (median = 0%) and those found through ham selection (median = 3.3%) possibly highlights this difference in sensitivity, although it should be also considered that deep hematomas are difficult to detect externally by visual inspection. Ham hematomas, despite their relatively high prevalence, showed a decrease from the values registered in this same plant during 2012 (4.3%) and 2013 (6.2%) [31], confirming the adoption of improved animal handling and other welfare measures declared by the slaughterhouse in 2017 [32].

Apart from veining, which showed minimum prevalence during summer, our seasonal trend for ham defects is not consistent with Arduini et al. [31], suggesting that other factors that are not strictly environmentally related should be taken into consideration to better understand the causes of these ham defects.

Despite a low annual median, veining represented a relatively frequent defect since it reached a maximum of 20.7%. The summer decrease of this defect could be related to the lower temperature of scalding water in this season (a lowering put into practice by the slaughterhouse), since in this season there is less hair on the carcass to remove. Nanni Costa et al. [33] previously highlighted the role of processing procedures, reporting that reducing time from carcass cutting to the start of ham chilling can contribute to a reduction in the prevalence of this defect. Beyond these process-related aspects, they also reported that resting pigs for 24h does not affect the prevalence of this defect, which is in contrast to the increase of veining that we found in overnight lairage batches. Nevertheless, the prolongation of pre-slaughter handling is a veining predisposing factor [34] and this can partially explain our result on overnight lairage. Further investigations about the causes are, however, necessary to confirm its seasonal trend and to develop more effective measures to prevent it.

PSE (pale soft and exudative meat), which is one of the most studied ham defects, despite a maximum prevalence of 4.9% was not detected in most of the batches, reflecting the satisfactory improvements achieved by genetics and management in regard to this defect.

Petechial haemorrhaging (meat spot defect) showed the lowest prevalence during winter, with spring that showed no statistical difference than the other seasons. Despite its median of < 1% it reached also relatively high prevalences (with a maximum of 6.8%). Overnight lairage was associated with a lower prevalence of this defect, suggesting a possible preventive role. Stunning methods have been reported as the main cause of these small blood spots spreading within the muscles [35], however the seasonality of this defect could be related to blood pressure variations due to vasodilation or vasoconstriction according to the temperature, since stunning procedures should remain constant throughout the year. The overnight lairage could contribute to making the animals quieter during the pre-stunning procedures, resulting in weaker muscular spasms after stunning and consequently in less capillary ruptures. Differences in pig heart rate between winter and summer (greater in winter) during loading and transport have previously been reported [18], suggesting that seasonal differences in blood pressure during pre-slaughter procedures could also exist. Farouk et al. [36] previously reported the role of blood pressure in petechial haemorrhaging, suggesting how arteriolar and venous dilatation (more associated with high environmental temperatures) could increase the prevalence of this defect.

When comparing heavy pigs intended for PDO hams and heavy pigs out of the circuit, the PDO animals had a higher prevalence for every ham defect. The different genetics, lean percentage and fat distribution could have determined this results, but further studies are needed to completely exclude any bias referable to the slaughterhouse operator sensitivity towards the different production types. Tail lesions were the only variable with a higher prevalence in non-PDO batches. In Italy the widespread use of tail docking and the common use of short docking (more than half of the tail docked) which further decreases the risk of tail biting [26] [37], can therefore contribute to this difference. Most non-PDO pigs are normally born in other European countries, and the possibility of different conditions in tail docking frequency and length could exist. In addition to the predisposing effect of barren environments, aberrant behaviours like tail biting are an indirect genetic collateral effect of selection for growth rate [38]. Non-PDO pigs have higher growth performance than PDO pigs (which cannot pursue the same carcass leanness to obtain the required quality for cured hams), and this could lead to differences in the expression of aberrant behaviours. In this sense it seems, however, to contrast with the higher prevalence of ear lesions in PDO than in non-PDO animals, although a substitution effect between tail and ear biting could exist. Overall, these results are difficult to interpret as a retrospective welfare indicator related to the specific production system.

## Conclusion

This extensive monitoring study on skin lesions detected on heavy pigs slaughtered at 170 kg highlights the importance of the slaughterhouse as an observation centre not only for health problems, but also for animal welfare, since such data can be collected and used to adopt preventive measures, that could positively affect also the product quality. The widespread prevalence of recent fighting-related lesions and traumatic/stress-related ham defects (which represent the majority of the total defects percentage) emphasises how all the procedures carried out from loading at the farm to stunning at the slaughterhouse are of critical welfare concern, suggesting that any improvement could have a profitable cost-benefit ratio.

The different results about the season influence on fighting-related lesions and ham defects, compared with those of similar studies, suggest that additional factors over-riding environmental parameters could be involved in their multifactorial causality. Overnight lairage was confirmed to be a predisposing factor for higher prevalence of fighting scratches, while its role in ham defect prevalence needs further investigation because our results on the affected variables are not well supported by the literature. On the basis of the collected data, it is not possible to state that fighting-related lesions are the main cause of traumatic and stress-related ham defects. However, it is likely that their role could be better assessed if a better observation point for scratches and hematomas on the thighs could be arranged at the slaughterhouse.

This study confirms how slaughterhouses may collect animal welfare information that is referable not only to the pre-slaughter phases but also to the rearing cycle. Ear lesions are suitable to be used as retrospective welfare indicator, while the effective use of tail lesions, would be possible only if tail docking were abandoned.
